# NFκB activation in differentiating glioblastoma stem-like cells is promoted by hyaluronic acid signaling through TLR4

**DOI:** 10.1038/s41598-018-24444-6

**Published:** 2018-04-20

**Authors:** Eva Ferrandez, Olga Gutierrez, David San Segundo, Jose L. Fernandez-Luna

**Affiliations:** 1 Genetics Unit Hospital Valdecilla and Instituto de Investigacion Valdecilla (IDIVAL), Av. Valdecilla s/n, 39008 Santander, Spain; 2 Immunology Service, Hospital Valdecilla and Instituto de Investigacion Valdecilla (IDIVAL), Av. Valdecilla s/n, 39008 Santander, Spain

## Abstract

We have previously described that the NFκB pathway is upregulated during differentiation of glioblastoma stem-like cells (GSCs) which keeps differentiating GSCs in a proliferative astrocytic precursor state. However, extracellular signals and cellular mediators of this pathway are not clear yet. Here, we show that TLR4 is a key factor to promote NFκB activation in differentiating GSCs. TLR4 is upregulated during differentiation of GSCs and promotes transcriptional activation of NFκB as determined by luciferase-reporter assays and expression of NFκB target genes. Downregulation of TLR4 by shRNAs or blockade with anti-TLR4 specific antibodies drastically inhibited NFκB activity which promoted further differentiation and reduced proliferation of GSCs. We found that hyaluronic acid (HA), a main component of brain extracellular matrix, triggers the TLR4-NFκB pathway in differentiating GSCs. Moreover, HA is synthesized and released by GSCs undergoing differentiation and leads to transcriptional activation of NFκB, which is inhibited following downregulation of TLR4 or blockade of HA synthesis. Thus, we have demonstrated that during the process of differentiation, GSCs upregulate TLR4 and release the TLR4 ligand HA, which activates the TLR4-NFκB signaling pathway. This strategy may efficiently be used by differentiating GSCs to maintain their proliferative potential and consequently their tumorigenic capacity.

## Introduction

Glioblastoma is a highly aggressive tumor with poor survival rates and treatment remains a challenge. Work in different types of cancer, including glioblastoma, suggest that cancer stem-like cells are resistant against radiotherapy and chemotherapy, which facilitates tumor recurrence^1^. A number of antitumor strategies induce DNA damage, or inhibit cell division and angiogenesis, mainly by small molecular inhibitors or blocking antibodies. Activation of DNA damaging mechanisms by genotoxic agents may induce apoptotic or senescent cell death^2^. Differentiation-inducing agents offer an alternative antitumor strategy and some compounds are able to increase the efficacy of chemotherapy or radiotherapy^3,4^. Based on this, strategies that promote differentiation and senescence provide therapeutic opportunities that need to be further explored.

There are many signaling pathways that promote oncogenic transformation or maintain the aggressiveness of tumor cells. Some of these pathways induce activation of NFκB, a transcription factor involved in many cellular processes including cell survival, proliferation and migration^5^. It has also been shown that inhibition of NFκB facilitates stemness^6^, and triggers proliferation of neural stem cells^7,8^. Proinflammatory signals like cytokines and pathogen-associated molecular patterns (PAMP) trigger an intracellular mechanism that leads to the activation of NFκB^9^. Several of these signaling pathways are initiated by activating cell surface receptors, including tumor necrosis factor receptor and Toll-like receptor (TLR) superfamilies. At present, 11 mammalian TLRs have been described. TLR proteins recognize PAMPS that include bacterial lipopolysaccharides and peptidoglycans or viral RNAs among others. Upon binding of the ligand, TLR proteins recruit a signaling adaptor protein, mainly MyD88, that activates a kinase cascade which ultimately promotes activation of the NFκB protein complex that is translocated to the nucleus to induce the expression of target genes. TLR4 is one of the most studied members of the TLR family and it has been involved in inflammation and resistance to virus, as well as in tumor microenvironment. TLRs, including TLR4, have been shown to be overexpressed in breast cancer^10^ and tumor cells from a wide variety of tissues^11^ suggesting that TLR activation may be an important event in tumor cell immune evasion. Activation of TLR4 in tumor cells promotes the synthesis of NFκB target genes, including IL-6 and IL1β, which results in resistance of tumor cells against cytotoxic lymphocytes. However, the role of the TLR4-NFκB signaling pathway during differentiation of cancer stem-like cells has been poorly studied.

Here we show that TLR4 is upregulated during differentiation of GSCs, which triggers the NFκB transcriptional pathway, avoiding terminal differentiation and maintaining proliferation. This cell behaviour is reversed following downregulation or inactivation of TLR4. We also show that TLR4 is activated by hyaluronic acid (HA) that is synthesized and secreted by differentiating GSCs.

## Materials and Methods

### Reagents

Blocking anti-TLR4 antibodies and control IgG2a (both from eBioscience-ThermoFisher, Waltham, MA) were mostly used at 0.5 μg/ml in cell cultures. HA fragments with a size distribution of 15–40 kDa (R&D Systems, Minneapolis, MN) were added at a concentration of 100 μg/ml when indicated. HA synthesis inhibitor 4-Methylumbelliferone (4-ME) (Sigma-Aldrich, St Louis, MO) was added to cell cultures at 2 mM. HA levels in cell culture supernatants were determined by an ELISA kit from Echelon Biosciences (Salt Lake City, UT).

### Primary tumor neurosphere cultures

GSCs were derived from patients with primary glioblastoma with wild-type IDH1 and EGFR amplification. All the results shown in figures have been obtained from a single cell culture, but in all cases similar data were replicated in one or two more GSC cultures. Neurosphere cultures were established as previously described^12^. Briefly, tumor cells were obtained following digestion of tissue samples and cultured in serum-free DMEM/F12 medium (Invitrogen, Carlsbad, CA) with growth factors containing human recombinant EGF (20 ng/ml EGF and 20 ng/ml bFGF, both from Sigma, St. Louis, MO). To induce differentiation, neurospheres were disaggregated into single cells that were cultured as a monolayer in the same medium but containing 10% FCS for the indicated time intervals as previously described^12,13^. For cell proliferation assays, neurospheres were disaggegated into single cell suspensions before cell growth was analysed at the indicated time intervals by using Alamar blue reagent (Life Technologies, Paisley, UK).

### Immunofluorescence

Briefly, differentiating GSCs were grown on 10 × 10 mm coverslips. Cells were then fixed in 3.7% formaldehyde and permeabilized with 0.5% Triton X-100. Then, cells were incubated with rabbit anti-GFAP (DAKO, Glostrup, Denmark) and anti-Ki67 (SP6, Thermo Scientific, Waltham, MA) or mouse anti-phospho histone H2AX (05–636, Millipore, Billerica, MA) antibodies. Texas red-conjugated or FITC-conjugated goat anti–rabbit or anti-mouse secondary antibodies (Jackson ImmunoResearch, Cambridgeshire, UK) were used for detection. For fluorescence quantification, markers were analyzed by using the AxioVision software (Carl Zeiss Inc.). Nuclear area was quantified using ImageJ software (NIH, Bethesda) on at least 100 DAPI-positive nuclei.

### Gene expression analyses

Gene expression analysis comparing neurosphere-forming GSCs and GSCs differentiated in the presence of 10% FCS for 4 days, was performed with the Human Genome U133 Plus 2.0 array (Affymetrix, Santa Clara, CA) as described^12^. Differential expression of all TLR family members included in the array was analyzed using the fold-change value. Gene expression was assessed by using primers for TLR4 (5′AGTTTCCTGCAATGGATCAAGG3) and (5′CTGCTTATCTGAAGGTGTTGCAC3), Map2 (5′TTCGTTGTGTCGTGTTCTCA3′) and (5′AACCGAGGAAGCATTGATTG3′), TNFα (5′CGGGACGTGGAGCTGGCCGAGGAG3′) and (5CACCAGCTGGTTATCTCTCAGCTC3′), IL-10 (5′CCGAGATGCCTTCAGCAGAG3′) and (5′CACATGCGCCTTGATGTCTG3′), IL-6 (5′AGTGAGGAACAAGCCAGAGC3′) and (5′GAGATGAGTTGTCATGTCCTGC3′), and β-Actin (5′GCGGGAAATCGTGCGTGACATT3′) and (5′GATGGAGTTGAAGGTAGTTTCGTG3′). Quantitative PCR was performed as previously described^14^.

### TLR protein expression analysis

Detection of the levels of TLR protein was achieved by using a TLR cell surface screening set (NOVUS, Littleton, CO) that included specific antibodies against TLR1, TLR2, TLR4, TLR5, TLR6 and TLR10. For each individual analysis, an isotype antibody was used for non-specific staining. PE-labeled secondary antibodies were then used and labelled cells were analysed by flow cytometry (FACSCalibur, BD Biosciences) using the ModFit software (Verity, Topsham, ME). TLR expression levels are shown as mean fluorescence intensity subtracting the corresponding isotype control.

### Transfection experiments and gene reporter assays

GSCs were co-transfected with 1 μg of pGL2-MAP2 promoter construct^12^ and 50 ng of pRSV-β-gal by nucleofection (Amaxa, Cologne, Germany). Cells were also transfected with 1 μg of pBVIx-Luc, a plasmid containing NFκB consensus sequences linked to the luciferase reporter gene. Luciferase activity was analysed by a dual-light reporter gene assay (Applied Biosystems). Results were normalized by cotransfection with pRSV-β-gal.

### Gene silencing

GSCs were dissociated from neurospheres and cultured at a density of 2 × 10^4^ cells per well in a 96-well plate. Five non-overlapping TLR4-specific or control scrambled shRNA-containing lentiviral particles were used (MISSION shRNA library, SIGMA) as indicated by the manufacturer. As a control of transduction efficiency, GSCs were infected with lentivirus containing shRNA to EGFP.

### Statistical analysis

Statistics were analyzed with the SPSS package (version 13.0). The Student t test was used to compare continuous variables as means ± SD, between two groups. The significance level was set at p < 0.05.

## Results

### TLR4 is upregulated during differentiation of GSCs and mediates activation of NFκB

**Figure 1:**
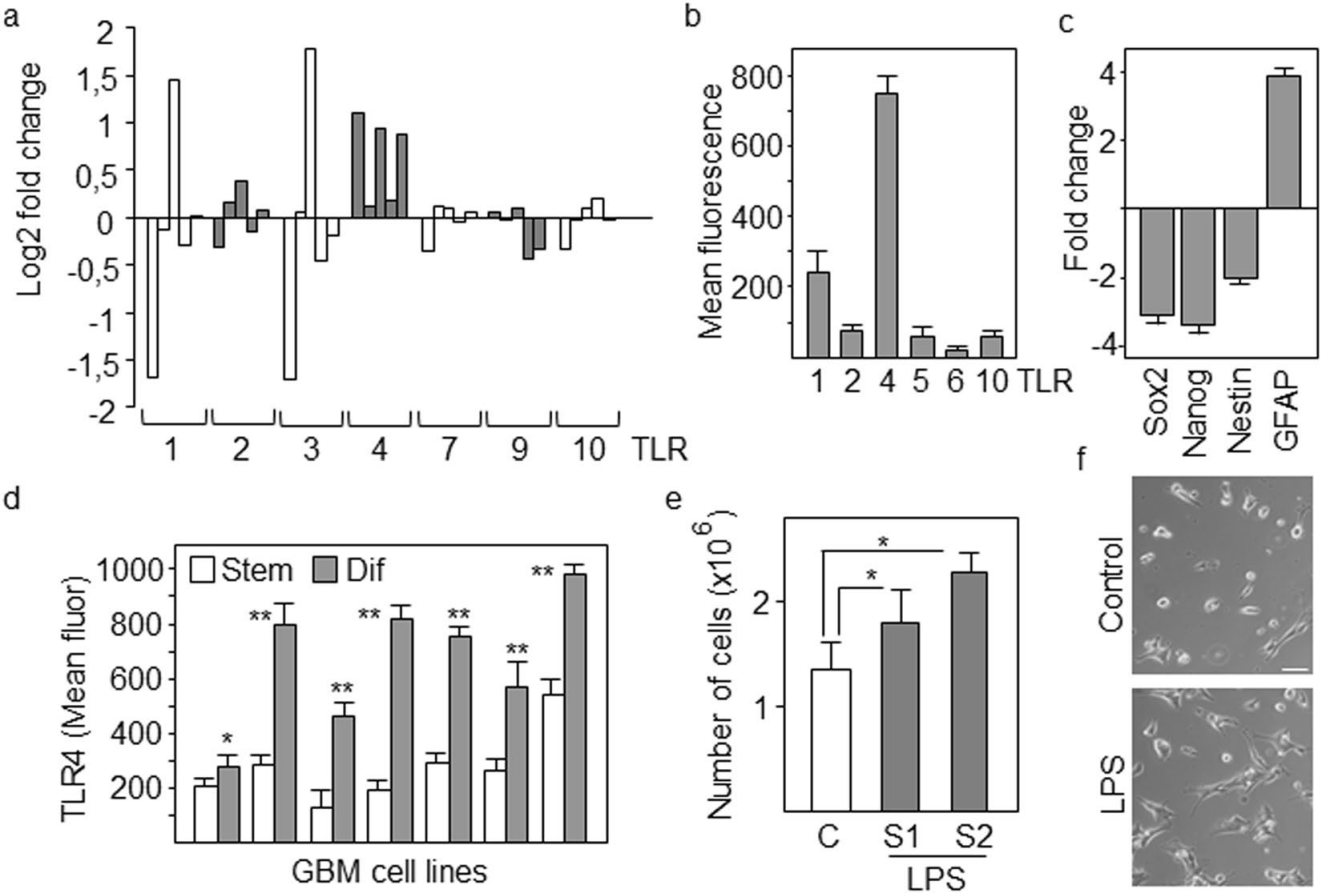
TLR4 is upregulated in differentiating GSCs. **(a)** Fold changes in the levels of TLR genes in differentiating compared with stem-like cells as assessed by gene expression microarray. **(b)** Flow cytometry analyses of TLRs. The expression in differentiating cells relative to stem-like cells is represented. (**c**) GSCs were induced to differentiate in the presence of serum for 4 days. The expression of progenitor (Nestin, Sox2, Nanog) and lineage (GFAP) markers was analyzed by qPCR. The expression levels were represented as fold changes of differentiating cells compared with GSCs. All markers showed significant differences (p < 0.01). (**d**) Expression levels of TLR4 protein in different GBM cell lines as determined by flow cytometry. Asterisks represent significant differences (*p < 0.05, **p < 0.01) compared to stem-like cells. **(e)** Proliferation and **(f**) Morphology of GSCs after 4 days of differentiation in the presence of LPS from E. coli strains O111:B4 (S1) and O55:B5 (S2). *p < 0.05. Histograms represent the mean ± SD of three independent experiments. Scale bar: 50 μm.

**Figure 2:**
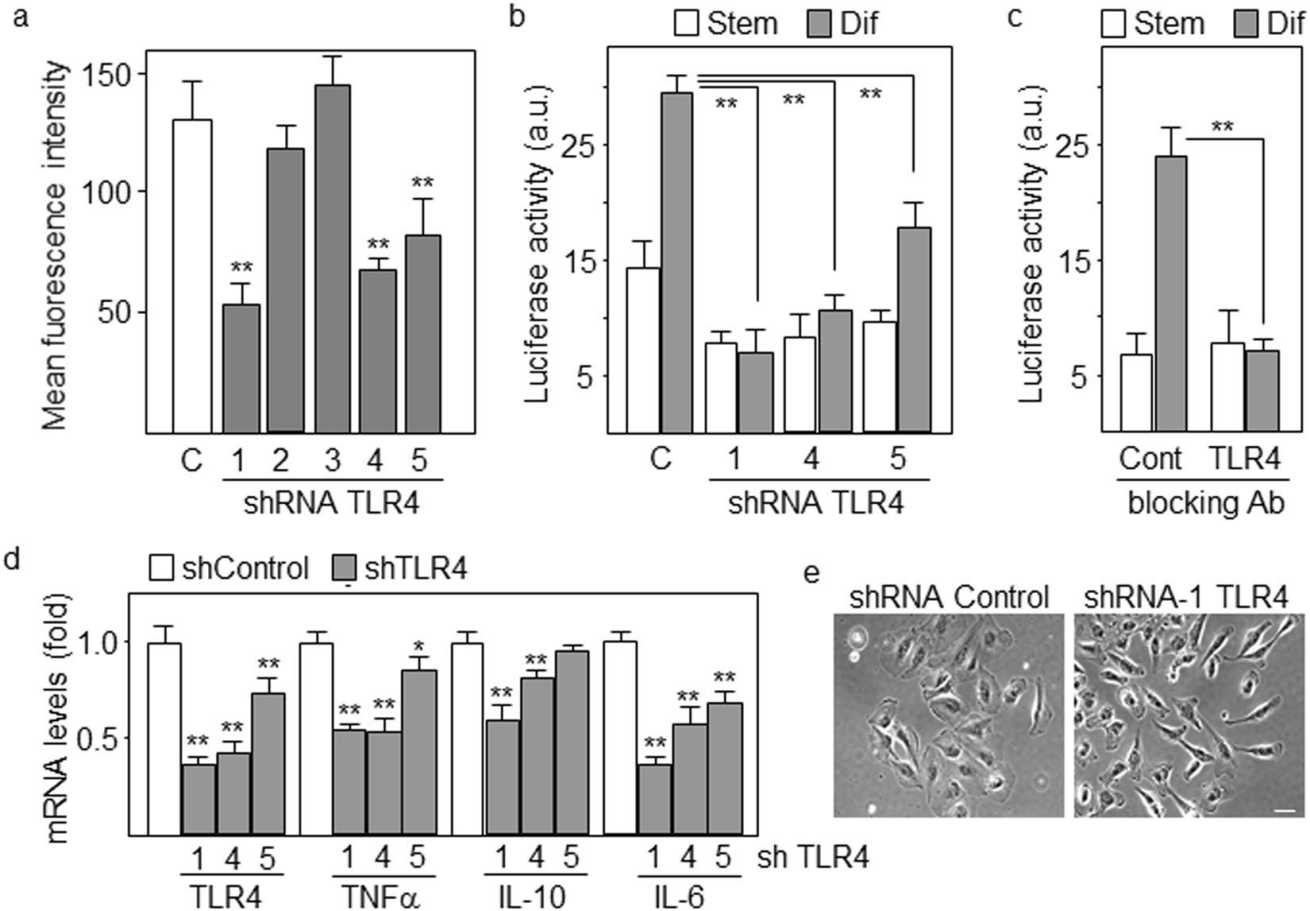
Downregulation of TLR4 blocks differentiation-induced activation of NFκB. **(a)** Protein levels of TLR4 are significantly reduced (**p < 0.01) in GBM cells with three out of five non-overlapping TLR4-specific shRNAs as determined by flow cytometry. (**b**,**c**) NFκB transcriptional activity following differentiation of GSCs transfected with TLR4-specific shRNAs (**b**) or cultured in the presence of TLR4 blocking antibodies (**c**). (**d**) Downregulation of TLR4 levels produced a decrease in the expression of NFκB target cytokines as determined by real-time PCR. (**e**) Morphological changes in GSCs with reduced levels of TLR4 after 4 days of differentiation. Scale bar: 50 μm. Histograms represent the mean ± SD of three independent experiments. **p < 0.01. a.u., arbitrary units.

We have previously shown that NFκB pathway is activated in differentiating GSCs (cultured in the presence of 10% FCS) avoiding terminal differentiation and promoting proliferation^12^. Consistently, GBM is characterized by poorly differentiated cancer cells with high mitotic activity^15^. The terms differentiating cells or cells undergoing differentiation, refer to a population of proliferative astrocyte precursors without stellate morphologies and with low levels of stem cell markers and increased expression of GFAP astrocytic marker. It is also well known that TLR signaling pathways promote activation of NFκB^16^. Our first goal was to study the expression of TLR family members in differentiating GSCs. Based on our microarray gene expression analyses of GSCs before and after differentiation for 4 days^12^, TLR4 was the only TLR member that increased its levels in all five cell lines tested (Fig. 1a). Flow cytometry further confirmed that TLR4 protein reached the highest levels following differentiation compared with the expression of TLR1, TLR2, TLR5, TLR6 and TLR10 (Fig. 1b). Downregulation of stem cell markers and increased levels of the astrocyte-specific marker GFAP confirmed the differentiation process (Fig. 1c). Then, TLR4 protein levels were studied in seven different GBM cell lines before (GSCs) and after differentiation and all of them upregulated TLR4 following differentiation (Fig. 1d). A well known ligand of TLR4 is the bacterial lipopolysaccharide (LPS), which has been shown to transduce proliferation signals in GBM cells^17,18^. As expected, TLR4 expressed in differentiated cells was functional, responding to LPS by increasing the proliferation of these cells (Fig. 1e and f). In order to study the contribution of TLR4 to NFκB activation, we transfected GSCs with five different TLR4-specific shRNAs. Three of these shRNAs significantly reduced the levels of TLR4 protein (between 40% and 60%) in differentiating cells as analyzed by flow cytometry (Fig. 2a). Downregulation of TLR4 dramatically decreased (about 5-fold with shRNA-1) the transcriptional activity of NFκB after differentiation as determined by using NFκB -luciferase reporter assays (Fig. 2b) and a similar result was obtained when we treated GSCs with a TLR4 neutralizing antibody that blocked signaling (Fig. 2c). NFκB pathway is able to promote the expression of a number of genes, including cytokines. As shown in Fig. 2d, downregulation of TLR4 reduced the expression of NFκB target genes TNFα, IL-10 and IL-6. Interestingly, low levels of TLR4 were associated with morphological changes in differentiating GSCs giving rise to a population of cells (more than 60 out of 100 cells analyzed as compared with 15% in control cells) with elongated morphologies and some stellate-like projections suggestive of a more differentiated state (Fig. 2e). This result is consistent with that we previously described by treating GSCs undergoing differentiation with an inhibitor of NFκB^12^.

### Blockade of TLR4 promotes differentiation and reduces proliferation of GSCs

**Figure 3:**
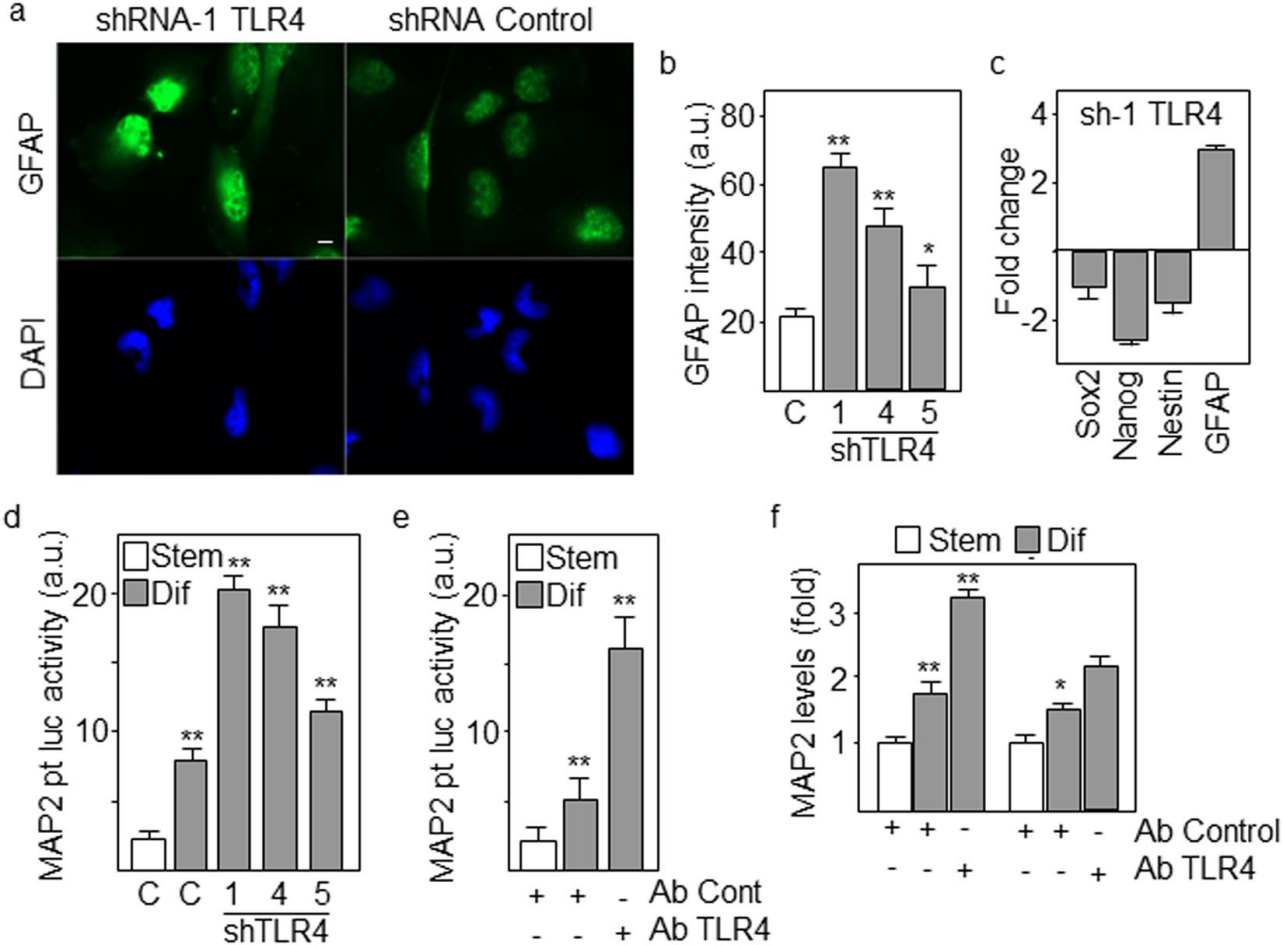
Downregulation of TLR4 accelerates differentiation of GSCs. (**a**,**b**) GSCs were transfected with the indicated TLR4-specific shRNAs and expression of GFAP after 8 days of differentiation was assessed by immunofluorescence. Fluorescence intensity was measured using ImageJ software. Scale bar: 20 μm. (**c**) Cells were transfected with control or TLR4-specific shRNAs and differentiated for 8 days. The expression of progenitor (Nestin, Sox2, Nanog) and lineage (GFAP) markers was analyzed by qPCR. The expression levels were represented as fold changes of cells transfected with TLR4 shRNA compared with control cells. All markers showed significant differences (p < 0.01). (**d**) Transcriptional activity of the MAP2 promoter in stem and differentiating cells following transfection with three TLR4-specific shRNAs. (**e**) Transcriptional activity of the MAP2 promoter in cells treated with blocking anti-TLR4 antibodies. (**f**) Expression levels of MAP2 mRNA in stem and differentiating cells treated with blocking antibody. Histograms represent the mean ± SD of three independent experiments. Asterisks in grey bars represent significant differences (*p < 0.05, **p < 0.01) compared to empty (control) bars. a.u., arbitrary units.

**Figure 4:**
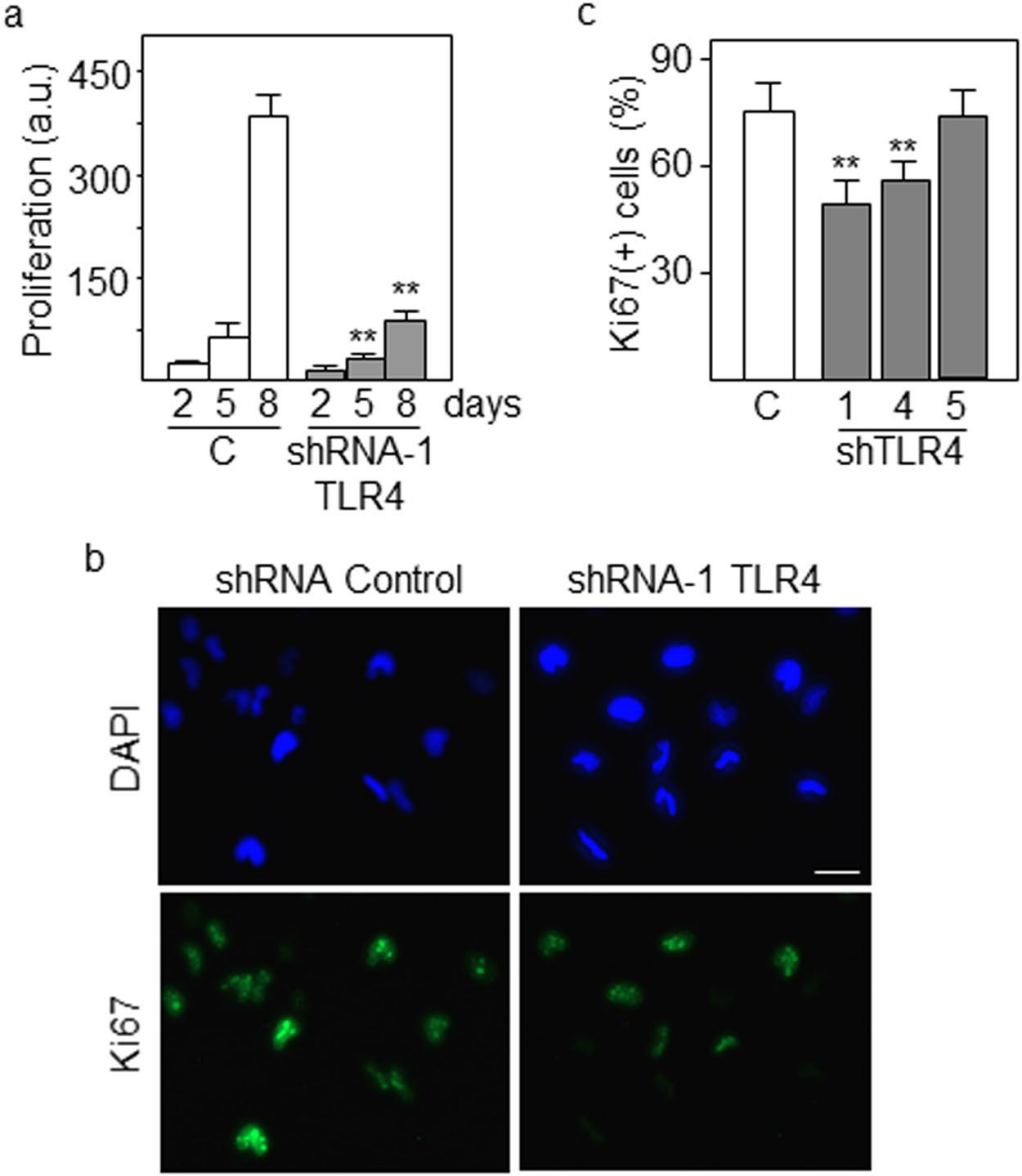
Proliferation of differentiating GSCs decreases following downregulation of TLR4. (**a**) GSCs were transfected with TLR4-specific shRNAs and proliferation was assessed at different time points of differentiation by using Alamar Blue reagent. a.u., arbitrary units. **(b)** Expression of the proliferation marker Ki67 and **(c)** quantification of Ki67-positive cells in GSCs with low levels of TLR4 following 5 days of differentiation. Cells were counterstained with DAPI. Histograms represent the mean ± SD of three independent experiments. Asterisks in grey bars represent significant differences (**p < 0.01) compared to empty (control) bars. Scale bar: 10 μm.

**Figure 5:**
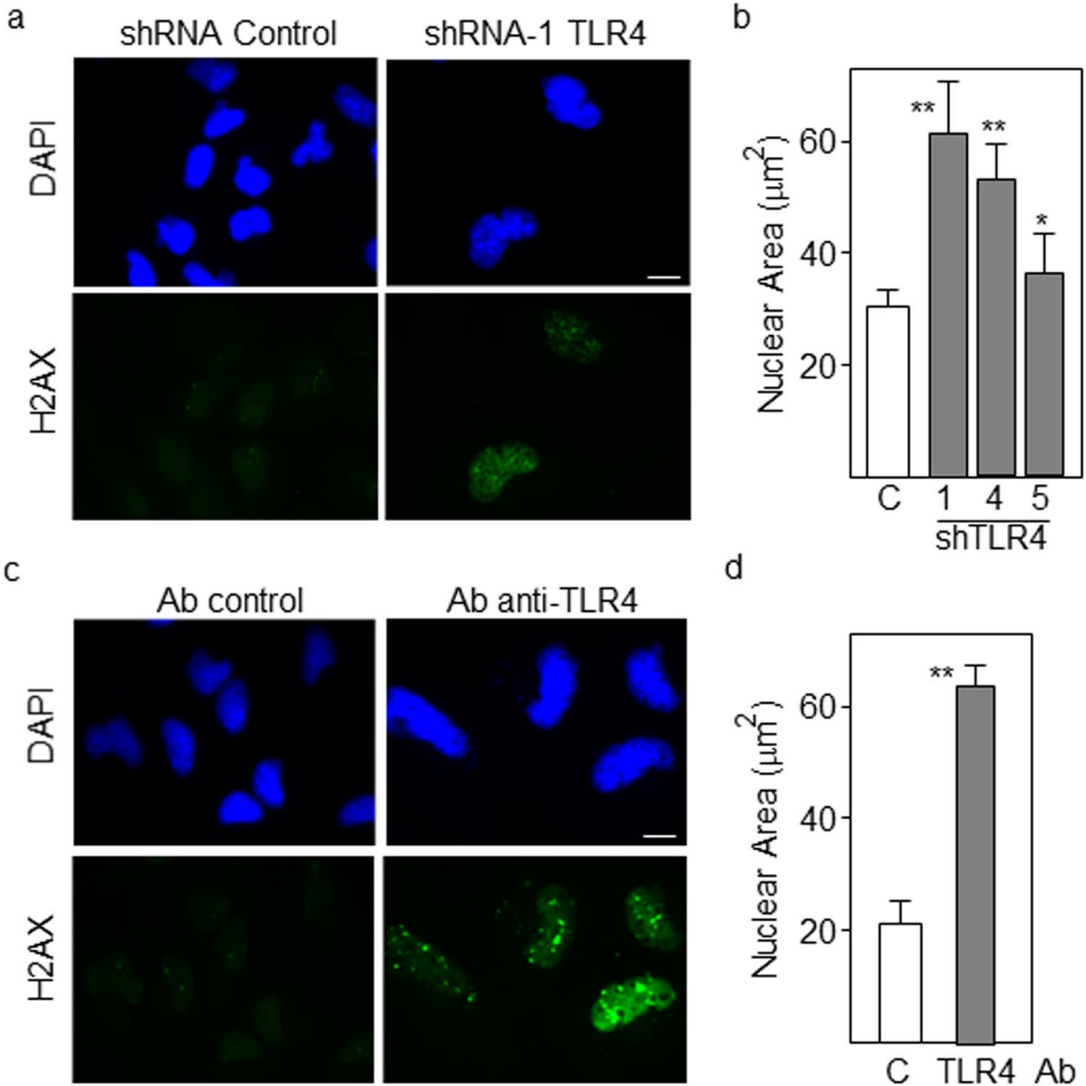
Differentiating GSCs acquire a senescent phenotype after downregulation of TLR4. **(a)** Detection of histone H2AX phosphorylation by immunofluorescence staining of GSCs transfected with TLR4-specific shRNAs or **(c)** treated with blocking anti-TLR4 antibodies after 5 days of differentiation. Histograms shown in **(b)** and **(d)** represent the nuclear area of DAPI-stained cells treated as described in (a) and (b) respectively. At least 20 nuclei were counted. Asterisks in grey bars represent significant differences (*p < 0.05, **p < 0.01) compared to empty (control) bars. Scale bar: 5 μm.

GSCs have the capacity to undergo differentiation to different neural lineages, mainly acquiring astrocytic (48–66%) and neuronal (22–45%) phenotypes^12^. In order to assess whether downregulation of TLR4 modified the differentiation pattern of GSCs, we first analyzed the expression of GFAP, an astrocytic differentiation marker, and found that low levels of TLR4 correlated with increased expression of GFAP (about 3-fold higher intensity with shRNA-1 relative to control) as determined by immunofluorescence (Fig. 3a and b). Transfection with the most efficient TLR4-specific shRNA reduced the levels of stem cell markers Sox2, Nanog and Nestin and increased the expression of GFAP in cells cultured under differentiation conditions (Fig. 3c). Additionally, we transfected GBM cells with the promoter region of the neuron-specific marker MAP2 linked to a luciferase reporter gene. Downregulation of TLR4 by shRNAs or functional blockade by specific anti-TLR4 antibodies highly increased the transcriptional activation of MAP2 in GSCs undergoing differentiation (Fig. 3d and e). In line with these results, the mRNA levels of MAP2 were also upregulated in differentiating cells following treatment with neutralizing anti-TL4 antibodies (Fig. 3f). Consistent with the acquisition of a mature phenotype following downregulation of TLR4, the proliferation capacity of GSCs was reduced more than 5-fold after 8 days of differentiation (Fig. 4a) as determined by the Alamar bioassay, a marker of metabolic activity frequently used to analyze cell proliferation. Another marker of proliferation, Ki67, was also significantly reduced in cells transfected with TLR4-specific shRNAs by counting the proportion of Ki67-positive cells (Fig. 4b and c). Two features of terminal maturation and senescence are the presence of megalonuclei^19^ and focal accumulation of phospho-histone H2AX at the sites of DNA double-strand breaks^20^. Consistently, when GSCs with low levels of TLR4 were differentiated for 8 days, the nuclear area was clearly increased (from about 30 μm^2^ to 60 μm^2^ with shRNA-1) and nuclei showed an accumulation of phospho-H2AX in approximately 24% of cells (Fig. 5a and b). Similar results but with more pronounced differences were obtained in cells treated with blocking anti-TLR4 antibodies for the same period of differentiation (Fig. 5c and d).

### TLR4 signaling is triggered by hyaluronic acid released from differentiating GSCs

**Figure 6:**
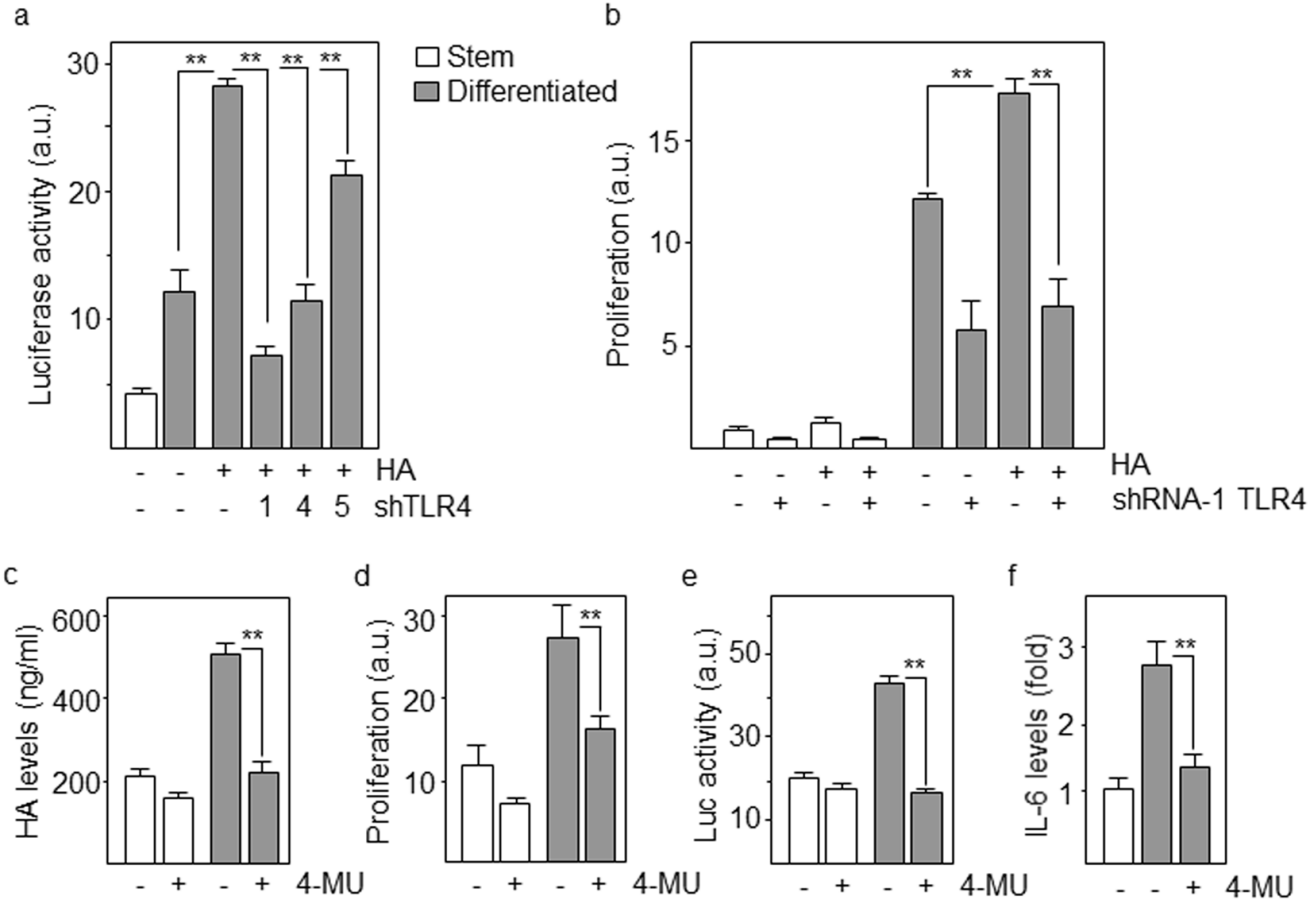
Hyaluronic acid triggers TLR4 signaling in differentiating GSCs. (**a**) Cells were cotransfected with the NFκB-luciferase reporter vector and TLR4-specific or control shRNAs in the presence or in the absence of hyaluronic acid (HA). Luciferase activity was determined after 48 h of incubation under stem or differentiation conditions. (**b**) Proliferation of cells in the presence of HA following downregulation of TLR4 as determined by Alamar blue. (**c**) HA levels present in the culture medium of stem and differentiating cells treated with the HA synthesis inhibitor, 4-MU by ELISA. (**d**) Proliferation of stem and differentiating cells following treatment with 4-MU. (**e**) Luciferase activity of cells transfected with the NFκB-reporter vector and treated with 4-MU. (**f**) Expression levels of IL-6 mRNA in differentiating GSCs treated with 4-MU by quantitative RT-PCR. Histograms represent the mean ± SD of three independent experiments. Asterisks represent significant differences (**p < 0.01). a.u., arbitrary units.

Once we have established that TLR4 is a main activator of the NFκB pathway during differentiation of GSCs, we asked about the ligand that triggers this signaling mechanism. Hyaluronic acid (HA) is a main component of the extracellular matrix in the brain^21^, and has been shown to signal through TLR4^22^. We showed that exogenous HA increases the NFκB transcriptional activity in differentiating GSCs and this activation was lost following downregulation of TLR4 by shRNAs (Fig. 6a). We have previously described that activation of NFκB in differentiating GSCs promotes proliferation and avoids terminal maturation^12^. In support of this, it has been shown in a number of cell lines that GSCs cultured under differentiation conditions are fast-growing^23^. Additionally, the absence of fully differentiated morphology of tumor cells in GSC xenografts^24^ and the high proliferation rate of astrocytic precursors^25^, indicate that GBM tumor cell populations comprise of proliferative progenitor cells that have not completed pathways toward astrocytic differentiation. We showed that incubation of GSCs undergoing differentiation in the presence of HA increased proliferation, which was drastically reduced when TLR4 was downregulated (Fig. 6b). Moreover, GSCs synthesized and secreted HA to culture medium and these levels were increased (about 2.5-fold) after 4 days of differentiation (Fig. 6c). We have also induced differentiation by growth factor withdrawal, which tipically results in spontaneous multi-lineage differentiation of GSCs^26^. Although differentiating cells exhibited less pronounced changes of differentiation markers, HA levels increased near 2-fold when compared with GSCs (1.7 ± 0.2 mean ± SD, p < 0.05) (data not shown). However, if we block the production of HA with 4-Methylumbelliferone (4-MU), a specific HA synthesis inhibitor used in many *in vitro* studies^27^, the secretion of HA by differentiating cells was reduced to levels similar to those of GSCs (Fig. 6c). Consistently, 4-MU reduced the proliferation capacity (Fig. 6d) and the NFκB transcriptional activity (Fig. 6e) of differentiating GSCs. To further confirm this result, we showed that the NFκB target gene IL-6 was upregulated in cells undergoing differentiation but its levels were drastically reduced in the presence of the HA synthesis inhibitor (Fig. 6f). Thus, HA produced and secreted during differentiation of GSCs triggers the TLR4-NFκB signaling pathway either in a paracrine or an autocrine manner.

## Discussion

GSCs have been identified as key cells for tumor initiation and recurrence, providing therapeutic opportunities^28^. We have previously demonstrated that canonical NFκB signaling, that is poorly activated in GSCs, is upregulated during differentiation of GSCs which allows cells to proliferate and avoid terminal differentiation and senescence^12^. This is consistent with previous work showing that canonical NFκB pathway has no or very little effect on GSCs^29^. In addition, activation of canonical NFκB has also been associated with mesenchymal transdifferentiation and radioresistance of a subset of GSCs^30,31^. However, the stimuli that trigger this activation in differentiating GSCs are not well defined. We showed that TLR4 was the only TLR member consistently upregulated in all differentiating GSC cultures established from surgical specimens of GBM patients. In line with this, it has been recently described that cancer stem cells isolated from patient-derived GBM xenografts showed a gradual increase in the expression of TLR4 under differentiation conditions^13^. TLR4 signaling results in activation of the NFκB pathway in astrocytes, microglia, neurons and neural progenitors^32^. Consistently, we demonstrated that TLR4 upregulation in differentiating GSCs was associated with increased NFκB transcriptional activity that was blocked by downregulating the levels of TLR4 or treatment with neutralizing TLR4 antibodies. Moreover, activation of the TLR4-NFκB axis in differentiating GSCs promoted proliferation of these cells and avoided terminal differentiation and senescence, a phenotype that characterizes GBM tumors. Experimental support of this phenotype comes from GSC xenograft models, that showed a clear absence of fully differentiated morphology indicating that tumor cell populations comprise of progenitor cells that have not completed astrocytic differentiation^24^. Previous data also showed that astrocytic precursors have a relatively high proliferation rate^25^. Consistent with our data, activation of the NFκB pathway is low in embryonic stem cells, but increases after differentiation^6,33^. However, it is not known whether this activation relies on a TLR4-mediated pathway. The low activation of canonical NFκB signaling in GSCs indicates that NFκB may be dispensable for survival and proliferation of stem cells, and this is consistent with a predominant accumulation of NFκB proteins in the cytoplasm of cells within neurospheres from embryonic mouse brain^7^. Recent data have shown that low TLR4 expression in GBM cancer stem cells allows these cells to survive by disregarding inflammatory signals^13^. Differentiated tumor cells would not have this adaptive advantage. However, on the other hand, a sustained activation of TLR4-NFκB will provide proliferation signals and some of the newly generated cells may develop biological mechanisms that enable them to migrate to more favorable microenvironments or survive to the immune attack. To this end, tumor heterogeneity is one of the most important hallmarks of GBM. Different cell subclones have been established from a GBM patient and all of them behave differently in terms of morphology, proliferation, response to treatment and aggressiveness in animal models^34^. NFκB has been shown to induce and also to inhibit differentiation indicating a broad spectrum of activities depending on the cell type^35–38^. We showed that blockade of TLR4-NFκB signaling by using genetic strategies or blocking antibodies accelerates maturation of differentiating GSCs. Previous data indicate a role of NFκB in terminal differentiation. For instance, activation of NFκB pathway induces cell division and inhibits differentiation of mammary epithelial cells^39^. Moreover, *in vivo* inhibition of NFκB signaling by using a small molecule induced sensescence of tumor cells in mice bearing GSC-derived tumors^12^.

At this point a key issue is how do GSCs undergoing differentiation activate the TLR4-NFκB pathway. It has been shown that hyaluronic acid (HA) is among the key organizers of the brain extracellular matrix^21,40^, and there are several examples showing that HA signals through TLR4^22,41^. We found that HA promotes activation of NFκB through TLR4 in differentiating GSCs when added to cell cultures. However, we observed an increase in NFκB activity in GSCs maintained under differentiation conditions without exogenous HA. Interestingly, we demonstrated that GSCs produce and secrete their own HA and this production is highly increased in differentiating cells. Blockade of HA synthesis abrogated the TLR4-NFκB pathway indicating that GSCs develop their own mechanism to assure activation of NFκB during the differentiation process. This is consistent with previous work showing that a human GBM cell line synthesized and secreted HA into the medium^42^. These authors also found that HA synthesis by glioma cells occurred during the proliferative phase, which is in line with our model of differentiating GSCs where upregulation of TLR4 and secretion of HA by intermediate precursor cells activate the NFκB transcriptional pathway. NFκB promotes proliferation by inducing the expression of growth factors such as IL1β or IL6^43^ and we showed that differentiating GSCs increased the levels of IL6 which were abrogated in the presence of a HA synthesis inhibitor. Thus, immature GBM cells may use this HA-TLR4-NFκB pathway as a mechanism to allow proliferation and avoid terminal differentiation and senescence. Although there are a number of studies demonstrating that HA signals via TLR4^41,44^ and even suggesting HA-TLR4 interactions^22^, another study claimed that HA was not a ligand but a regulator of TLRs^45^. Two relevant issues may help explain these conflicting results. First, those studies that suggest a ligand-receptor scenario used HA fragments with molecular size above 100 kDa, whereas the study arguing that HA is a TLR4 regulator used HA fragments below 3 kDa and this is an important issue as HA biological activities differ widely depending on the fragment sizes^46^. Additionally, all these works used different cell models, including glomerular mesangial cells, macrophages, dendritic cells and endothelial cells which may have different responses to HA. Further biochemical studies will be needed to undoubtedly identify HA as a cognate TLR4 ligand. It would also be interesting to study the relevance of the HA-TLR4 pathway in other *in vitro* or *in vivo* models that provide a more physiological context. In this regard, 3D cultures such as the spheroid model have been used for evaluating drug efficay or response to antiangiogenic conditions^47–49^. However, the cellular heterogeneity of GBM may need more complex *in vitro* systems or *in vivo* xenograft models to more closely mimic the native tumor microenvironment.

Overall, we have demonstrated that TLR4 is upregulated during the differentiation process of GSCs and that TLR4 promotes activation of the canonical NFκB pathway in these cells. TLR4-NFκB signaling is mainly orchestrated by HA that is synthesized and secreted by differentiating GSCs. Our results provide new findings for therapeutic opportunities aimed to promote terminal differentiation of GSCs through inhibition of the NFκB signaling pathway.
